# Optimization of panoramic radiographic dose for third molar dental age assessment

**DOI:** 10.1007/s00414-026-03747-8

**Published:** 2026-04-20

**Authors:** Carolina de Paula Rossetto Lisboa, Ademir Franco, Anne Caroline Costa Oenning, João Pedro Rangel-Coelho, Monikelly do Carmo Nascimento, José Luiz Cintra Junqueira, Mariana Quirino Silveira Soares

**Affiliations:** 1 Division of Forensic Odontology, São Leopoldo Mandic, Campinas, SP Brazil; 2 Department of Radiology, University of Campinas (UNICAMP), Piracicaba, SP Brazil; 3 Division of Oral Radiology, São Leopoldo Mandic, Campinas, SP Brazil

**Keywords:** Age estimation by teeth, Radiology, Forensic dentistry, Panoramic radiography

## Abstract

This study aimed to assess the influence of reduced exposure parameters on the reproducibility of two radiographic dental age assessment methods in panoramic radiographs: the staging system (GHK) proposed by Gleiser and Hunt (1955) modified by Köhler et al. (1994) and the metric method by Cameriere et al. (2008) (I3M). Five phantoms were prepared using dry human skulls and subjected to three different radiographic image acquisition protocols with variations in peak kilovoltage (kVp) and tube current (mA), resulting in different absorbed dose levels: Protocol 1 (70 kVp, 12.5 mA), Protocol 2 (66 kVp, 8 mA), and Protocol 3 (66 kVp, 3.2 mA). A total of 57 cropped digital images of third molars were obtained and evaluated by 10 trained examiners at two different times. Intra-observer agreement was assessed using Fleiss’ weighted Kappa coefficient for the GHK method and the intraclass correlation coefficient (ICC) for I3M. The results showed substantial to good agreement for both methods, with high mean kappa and ICC values and no significant statistical differences among the protocols. In the dichotomous classification of legal adulthood (< 18 or ≥ 18 years) using the I3M, the lowest-dose protocol performed equivalently to the standard protocol. Considering the current study settings, reducing exposure parameters did not compromise the reproducibility of radiographic third molar analysis for dental age assessment methods. This outcome supports the use of low-dose image acquisition protocols as a potentially viable and (bio)ethical alternatives in forensic contexts.

**Supplementary Information:**

The online version contains supplementary material available at 10.1007/s00414-026-03747-8.

## Introduction

The assessment of the legal age of 18 years has become an increasingly common requirement in forensic dental practice, with direct implications for judicial, migratory, and humanitarian contexts [1, 2]. More specifically, adolescents and young adults can be subjected to radiographic examinations in cases of immigration, adoption, asylum application, or criminal/civil liability [3–6].

Among the established methods for this purpose are those proposed by Gunst et al. [7] (2003) and Cameriere et al. [8] (2008). The Gunst method employs a morphological staging system developed by Gleiser and Hunt [9] (1955) and modified by Köhler et al. [10] (1994). The system classifies third molars into developmental stages from 0 to 10 based on the degree of crown and root mineralization [10]. GHK third molar classification has already been applied in dental age assessment studies worldwide, including samples from Malaysia [11], Russia [12], United Arabic Emirates [13] and Brazil [14, 15]. The Cameriere’s method (I3M) [8], on the other hand, is based on a metric approach, calculating the ratio between the linear measurements of the apical opening(s) and the tooth length to estimate age. The method yields a dichotomous classification based on a cut-off value that distinguishes individuals below or above the legal threshold of 18 years [8]. Its application also extends to several countries [16], including Brazil [17].

Panoramic radiography is widely used in these contexts because it provides a comprehensive view of the dentomaxillofacial structures with lower exposure compared to cone-beam CT [18]. However, exposure to ionizing radiation, even at reduced levels, has raised ethical and biological concerns, particularly for individuals in vulnerable situations [19–21]. For this reason, the ALADAIP principle (As Low As Diagnostically Acceptable being Indication-oriented and Patient-specific) has recommended radiological-related practices with the lowest possible dose that could still maintain diagnostic quality. This principle derives from the well-established ALARA concept (As Low As Reasonably Achievable) and emphasizes the need to adjust exposure parameters according to both the diagnostic indication and the patient’s induvial characteristics [22, 23].

In this context, it is worth mentioning that in the medical use of radiographic image acquisition, some technical parameters such as peak kilovoltage (kVp) and tube current (mA) can be adjusted, possibly reducing radiation dose without necessarily compromising diagnostic quality [24]. Martins et al. (2021) [25] demonstrated that dose reduction in panoramic radiographs is practicable and may not impair the diagnostic performance of healthcare professionals.

To date, however, there are no studies investigating whether the reduction of exposure parameters in panoramic radiographs affects the performance of third molar metric and non-metric analyses for dental age assessment. This gap is relevant considering the increasing number of forensic contexts in which the investigation of legal majority is required with safety and ethical responsibility [26–28].

This study aimed to compare two radiographic techniques for third molar analyses on panoramic radiographs obtained with different settings of exposure parameters. The null hypothesis is that variations in exposure parameters and radiation dose do not affect the diagnostic performance of forensic odontologists in third molar assessment.

## Materials and methods

### Study design and ethical aspects

This is a prospective, controlled observational study with experimental simulation, approved by the Institutional Research Ethics Committee (CAAE: 76808923.3.0000.5374). The study was performed with radiographic images obtained from dry human skull phantoms. The study was designed and reported in accordance with the STROBE guidelines (Strengthening the Reporting of Observational Studies in Epidemiology) (Suppl 1).

### Phantom construction and image acquisition

Five models containing 19 third molars were prepared. The phantoms, composed of dry human skulls, were previously selected based on the presence of third molars, mandibles, hyoid bones, and the first four cervical vertebrae, sourced from the Anatomy Laboratory collection. The presence of third molars and their developmental stages were confirmed through periapical radiographs evaluated by a maxillofacial radiologist and a forensic odontologist. Skulls presenting lesions or fractures were excluded.

The structures were assembled in a standardized anatomical arrangement and covered with a homogeneous 3 mm thick layer of utility wax enveloping all bony structures to simulate soft tissues.

**Fig. 1:**
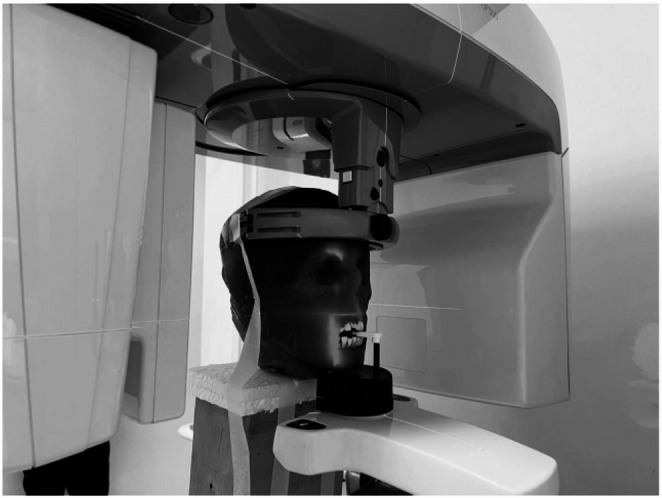
Model positioned in the panoramic radiographic unit

**Fig. 2:**
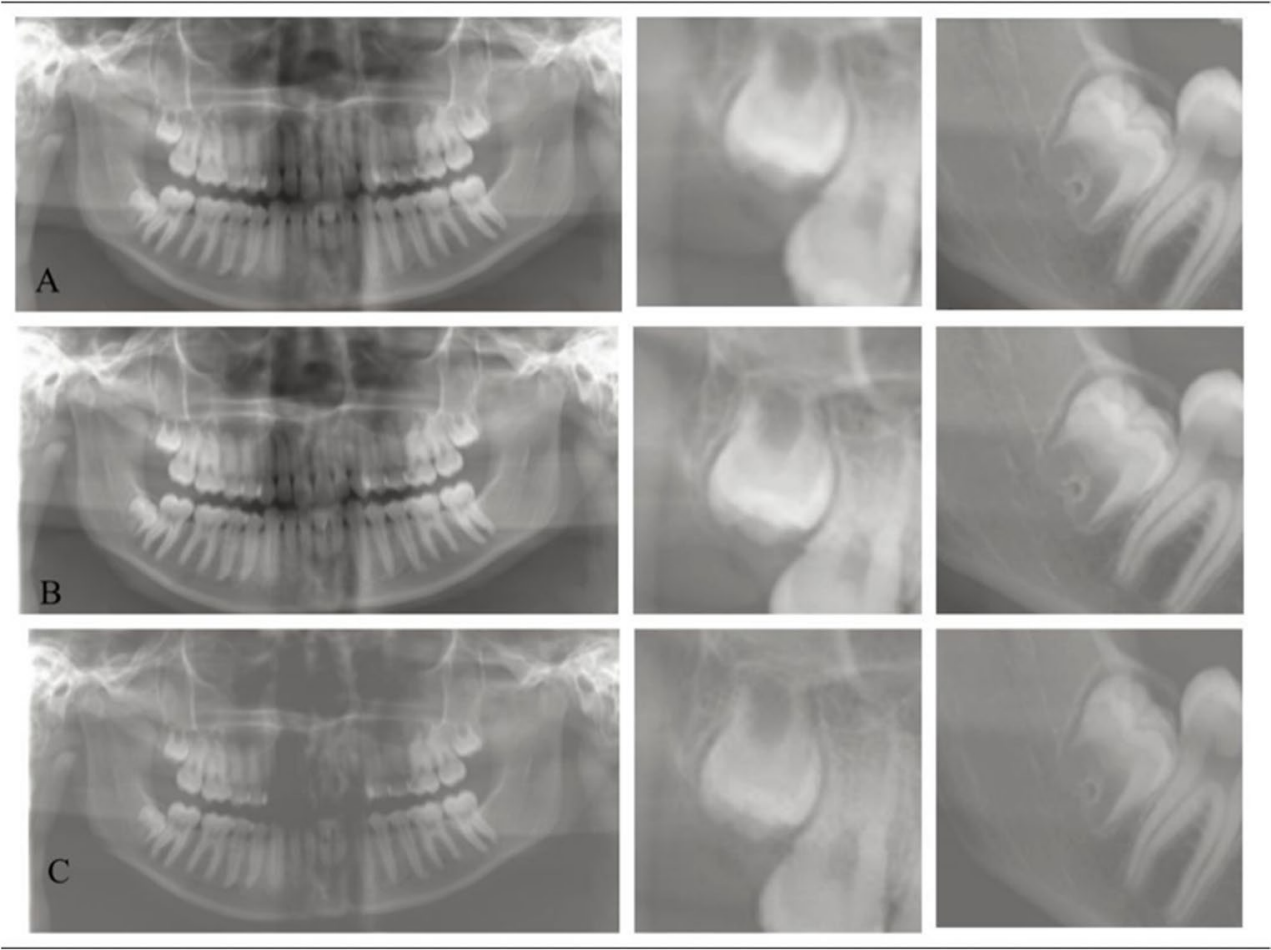
Radiographs and cropped images obtained with the three acquisition protocols. A: 70 kVp, 12.5 mA, 3393 µGy; B: 66 kVp, 8 mA, 1841 µGy; C: 66 kVp, 3.2 mA, 720 µGy

Images were acquired using a panoramic radiography device, the OP300 Maxi Unit (Instrumentarium, Tuusula, Finland) (Fig. 1). Three acquisition protocols (Fig. 2) were applied to the five phantoms, with different combinations of peak kilovoltage (kVp) and tube current (mA), as well as corresponding absorbed dose levels in the salivary glands, as described by Martins et al. (2021) [14] Protocol 1 (reference): 70 kVp, 12,5 mA, 3393 µGy; Protocol 2: 66 kVp, 8 mA, 1841 µGy; Protocol 3: 66 kVp, 3,2 mA, 720 µGy.

The exposure time was kept constant at 16 s. All acquisitions were performed using manual exposure mode with manufacturer-defined fixed presets. After acquisition, the images were exported in raw form as uncompressed TIFF format using Cliniview software (DEXIS, Hatfield PA).

Figure 2 shows the cropped radiographic images obtained from the three evaluated protocols. Image A corresponds to the highest dose protocol and displays the tooth with well-defined contours, sharply outlined root, and a clearly visible apical region, which favors both the classification via GHK and Cameriere’s metric method. In image B, obtained with the intermediate dose protocol, image quality remains satisfactory, although a slight loss of contrast and smoothing of root margins can be observed, nevertheless, critical structures such as the apex and root length remain visible. Image C, corresponding to the lowest dose protocol, shows lower optical density, increased noise, and more diffuse contours. The overall tooth morphology is preserved.

### Participants

Ten examiners with a degree in Dentistry and prior experience in Forensic Odontology participated in the study. All were invited, voluntarily consented to take part, and signed the informed consent form. The inclusion criteria were familiarity with radiographic methods for age estimation and current professional activity. Examiners who failed to complete the analysis of all images or who lacked proficiency with computer-based tools for image measurement and visualization were excluded.

### Application of age estimation methods

Each examiner received detailed written instructions on the GHK [9, 10] and I3M [8] third molar assessment techniques. A data recording sheet was also provided to register their staging and metric analyses. The images were randomized and distributed to the examiners over five consecutive weeks, so that one image corresponding to each of the five phantoms could be analyzed per week. For standardization purposes, all the phantoms were considered males. Examiners performed the measurements individually, independently, and blindly (without prior knowledge of the acquisition protocol used for each image). To reduce memorization of the radiographs (in addition to the interval between analyses) and biasing from other teeth, the images were cropped to include only the third molar and the adjacent second molar. Each third molar was analyzed under the three different acquisition protocols. Thus, a total of 57 images (19 teeth × 3 protocols) were analyzed by each participant.

### GHK and Cameriere’s techniques

**Fig. 3:**
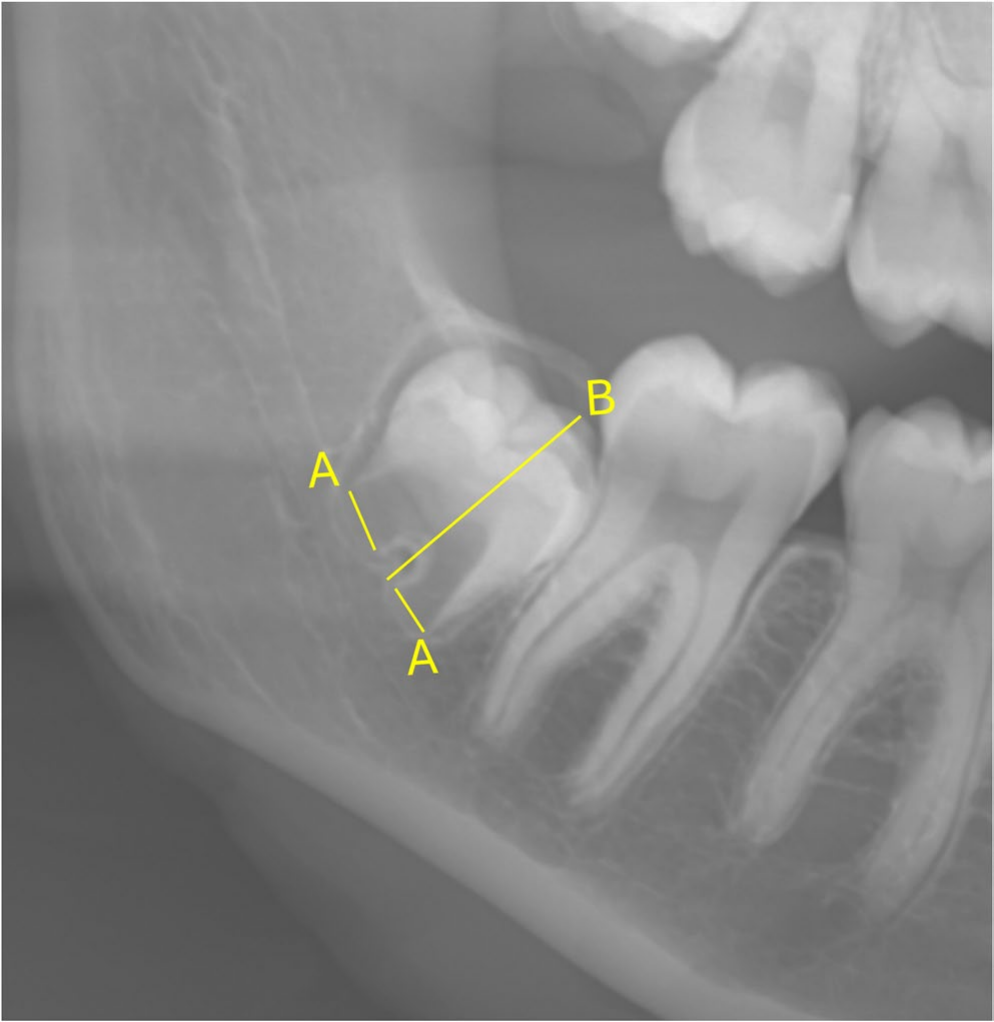
Illustration of the technique by Cameriere et al., 2008

GHK consists of the morphological and radiographic assessment of third molar dental development. Each third molar is classified on an ordinal scale from 0 to 10, according to the degree of crown and root mineralization and apical closure. Early stages (0 to 4) represent crown formation and initial root development phases, while intermediate stages (5 to 7) correspond to partial root formation with open apices. Final stages (8 to 10) indicate completion of development, with fully formed roots and progressive apical closure [10]. The Cameriere method is based on the ratio between the sum of the apical openings of the lower left third molar and the tooth length, called the third molar maturity index (I3M). The cutoff point used was I3M = 0.08, as originally proposed by Cameriere et al. (2008) [8]. Values below this threshold classify the individual as an adult (using 18 years as the legal age reference) [8] (Fig. 3).

### Pilot study

Before the main phase of the research, a pilot study was conducted to verify the feasibility of the image acquisition protocols, the adequacy of the selected radiographic cropping, and the examiners’ understanding of the analyses. Four dentists were recruited for the pilot study (following the preestablished eligibility criteria). They received five digital folders containing the cropped radiographs of third molars and instructions to analyze the third molars and register their staging and measurements. Image analysis by these specialists was conducted in two separate sessions, which contributed to the validation to the final adjustments of the methodological procedures adopted in the main phase of the study.

### Statistical analysis

The sample size calculation for determining the number of phantoms and evaluators was performed using Stata 18.0 software, based on parameters described by Martins (2021) [25], considering: for GHK, an expected Kappa of 0.80, precision of 0.10, and 10% positive classifications, resulting in a minimum of 19 assessments per examiner; for I3M, an expected ICC of 0.80, standard error of 0.10, and statistical power of 80%, resulting in a minimum of 18 observations per examiner.

Intra-examiner agreement was assessed separately for each method: for I3M (continuous variable), the Intraclass Correlation Coefficient (ICC) was used; for GHK (ordinal classification), the Fleiss’ weighted Kappa coefficient was applied. Individual coefficients and overall averages between protocols 1 × 2 and 1 × 3 were calculated. Besides the mean values, 95% confidence intervals were estimated using the bootstrap method with 1,000 iterations. Overlapping 95% confidence intervals were used to assess the presence of statistically significant differences. Agreement between the methods for classifying individuals regarding legal majority (< 18 or ≥ 18 years) was calculated using the Kappa coefficient for both Cameriere and GHK methods. For interpreting Kappa values, the criteria proposed by Landis and Koch [18] were adopted, classifying agreement levels as follows: poor (< 0.00), slight agreement (0.00–0.20), fair agreement (0.21–0.40), moderate agreement (0.41–0.60), substantial agreement (0.61–0.80), and almost perfect agreement (0.81–1.00). The interpretation of Intraclass Correlation Coefficients (ICC) followed the criteria proposed by Koo and Li [19], which classify reliability as low (ICC < 0.50), moderate (0.50–0.75), good (0.75–0.90), and excellent (ICC > 0.90). The significance level was set at 5%. All analyses were performed using Stata 18.0 (StataCorp LLC, College Station, TX, USA).

## Results

A total of 57 digital images, obtained from 19 third molars belonging to five phantoms and subjected to three different acquisition protocols, were evaluated. Each image was independently assessed by 10 examiners, resulting in 1,140 classifications for each method.

For GHK, the comparison between protocols 1 and 2 (Table 1) led to agreements from 91.0% to 98.5%, while the Fleiss’ weighted Kappa coefficient showed values between 0.687 and 0.958. The mean Kappa coefficient was 0.799, with a 95% confidence interval from 0.744 to 0.854, indicating agreement levels ranging from substantial to almost perfect among the examiners.

**Table 1:** Agreement of GHK staging technique between protocols 1 and 2

Evaluator	Expected agreement by chance	Observed agreement	Kappa	95% CI
1	71.2%	91.0%	0.687	0.323; 0.767
2	64.5%	94.7%	0.852	0.655; 0.922
3	68.1%	93.2%	0.788	0.667; 0.839
4	64.5%	94.7%	0.852	0.302; 0.904
5	67.6%	95.5%	0.861	0.751; 0.934
6	67.3%	95.5%	0.862	0.767; 0.927
7	66.2%	91.0%	0.733	0.554; 0.758
8	70.0%	92.5%	0.749	0.543; 0.876
9	62.4%	86.8%	0.650	0.593; 0.751
10	64.0%	98.5%	0.958	0.908; 0.972
Mean		93.3%	0.799	0.744; 0.854

CI: confidence interval

In the comparison between protocols 1 and 3 (Table 2), the observed agreement ranged from 89.5% to 96.5%, with Kappa coefficients varying between 0.649 and 0.903. The mean Kappa value was 0.796 (95% CI: 0.747–0.845), representing a level of agreement similar to that observed between protocols 1 and 2.

**Table 2:** Agreement of GHK staging technique between protocols 1 and 3

Evaluator	Expected Agreement by Chance	Observed Agreement	Kappa	95%IC
1	73.8%	92.8%	0.724	0.605; 0.801
2	67.6%	94.7%	0.838	0.656; 0.925
3	64.0%	96.5%	0.903	0.780; 1.000
4	67.6%	94.7%	0.838	0.741; 0.901
5	68.3%	90.4%	0.696	0.590; 0.750
6	68.6%	94.7%	0.832	0.751; 0.890
7	66.8%	92.5%	0.774	0.610; 0.897
8	70.0%	89.5%	0.649	0.600; 0.796
9	62.2%	93.9%	0.838	0.659; 0.965
10	65.8%	95.6%	0.872	0.820; 0.931
**Mean**		93.5%	0.796	0.747; 0.845

CI: confidence interval

For I3M, ICC values between protocols 1 and 2 ranged from 0.605 to 1.000, with an average of 0.819 (95% CI: 0.727–0.912), demonstrating good agreement (Table 3).

**Table 3:** Agreement of the I3M metric technique between protocols 1 and 2

Examiner	ICC	95%CI
1	0.987	0.966; 0.995
2	1.000	
3	0.605	0.126; 0.859
4	0.639	0.281; 0.842
5	0.769	0.501; 0.903
6	0.984	0.959; 0.994
7	0.790	0.541; 0.913
8	0.824	0.606; 0.928
9	0.950	0.868; 0.982
10	0.644	0.277; 0.849
Mean	0.819	0.727; 0.912

ICC: Intraclass correlation coefficient; CI: confidence interval.

In the comparison between protocols 1 and 3 (Table 4), individual ICCs ranged from 0.636 to 1.000, with a mean value of 0.821 (95% CI: 0.721–0.920), demonstrating good agreement. There was no significant difference between the mean values of the two comparisons, which was supported by the overlap of the confidence intervals.

**Table 4:** Agreement of the I3M metric technique between protocols 1 and 3

Examiner	ICC	95% CI
1	0.679	0.334; 0.866
2	1.000	1.000; 1.000
3	0.672	0.257; 0.880
4	0.658	0.311; 0.852
5	0.702	0.384; 0.873
6	0.993	0.984; 0.997
7	0.636	0.276; 0.841
8	0.963	0.907; 0.986
9	0.972	0.924; 0.990
10	0.930	0.826; 0.973
Mean	0.821	0.721; 0.920

CI: confidence interval.

### Agreement regarding legal adulthood classification

For I3M comparison between protocols 1 and 2 was performed considering the dichotomous classification of legal majority (< 18 or ≥ 18 years). The mean observed agreement among the examiners was 89.7%, with a mean Kappa coefficient of 0.755 (95% CI: 0.505–1.000). For the comparison between protocols 1 and 3, the results were similar, with a mean observed agreement of 90.9% and a mean Kappa of 0.767 (95% CI: 0.516–1.000). Perfect agreements (Kappa = 1.0) were recorded for at least three examiners in both comparisons (Tables 5 and 6).

**Table 5:** Agreement between protocol 1 and protocol 2 for classifying individuals regarding legal majority using I3M

Examiner	Expected Agreement by Chance	Observed Agreement	Kappa	95% CI
1	55.6%	100%	1.0	-
2	53.5%	100%	1.0	-
3	48.4%	81.2%	0.636	0.291; 0.982
4	57.4%	94.4%	0.87	0.623; 1.0
5	60.7%	89.5%	0.732	0.394; 1.0
6	50.7%	68.4%	0.36	-0.053; 0.7
7	54.8%	94.7%	0.883	0.663; 1.0
8	52.9%	78.9%	0.553	0.176; 0.93
9	56.2%	89.5%	0.759	0.453; 1.06
10	100%	100%	-	-
Mean		89.7%	0.755	0.505;1.0

CI: confidence interval

**Table 6:** Agreement between protocol 1 and protocol 3 for classifying individuals regarding legal majority using I3M

Examiner	Expected Agreement by Chance	Observed Agreement	Kappa	95% CI
1	59.3%	88.9%	0.727	0.384; 1.0
2	53.5%	100%	1.0	-
3	51.6%	93.8%	0.871	0.628; 1.0
4	57.4%	94.4%	0.87	0.623; 1.0
5	60.7%	89.5%	0.732	0.394; 1.0
6	54.8%	73.7%	0.417	-0.009; 0.843
7	56.8%	100%	1.0	-
8	54.8%	73.7%	0.417	-0.009; 0.843
9	58.7%	94.7%	0.872	0.631; 1.114
10	100%	100%	-	-
Mean		90.9%	0.767	0.516; 1.0

CI: confidence interval.

## Discussion

This study investigated the influence of different exposure parameters in panoramic radiography on third molar evaluation for dental age assessment using two well-established forensic methods, the GHK staging system and the I3M metric approach. The reduction of exposure parameters did not compromise intra-examiner reproducibility, as agreement coefficients remained within good to substantial ranges for both methods [29–31]. The reproducibility of I3M was consistent with previous studies across different populations [17, 32–36], while GHK also demonstrated strong agreement, likely supported by examiner training and standardized interpretative guidelines, which are known to reduce subjectivity and improve reliability in radiographic assessments [37–40].

Agreement levels were also influenced by the complexity of the diagnostic task. GHK relies on an ordinal scale with ten developmental stages, requiring discrimination of subtle morphological differences, particularly between advanced stages with nearly complete apical formation. This subjectivity is well recognized in the literature and contributes to classification variability [7, 12, 39]. Additionally, the Fleiss’ weighted Kappa coefficient used for GHK is sensitive to category distribution, potentially yielding lower values even when observed agreement is high [39, 40].

Differences between staging-based and measurement-based approaches are also relevant. Linear measurements such as I3M may be more sensitive to reduced image sharpness, as smoothed edges and less distinct landmarks can affect measurement accuracy. Nevertheless, the consistently high reproducibility observed across exposure protocols indicates that the degree of image alteration introduced by dose reduction was not sufficient to compromise the diagnostic task under investigation. Furthermore, while GHK can be applied to maxillary and mandibular third molars [9], I3M was originally developed for the mandibular left third molar only [8], and the inclusion of maxillary teeth may have introduced additional variability [41].

Importantly, this study did not aim to assess age estimation accuracy, but rather the reproducibility of third molar analysis under different acquisition conditions. The consistently high agreement observed suggests that diagnostic reliability was preserved despite dose reduction, supporting the feasibility of task-based dose optimization in panoramic imaging. These findings are consistent with previous studies demonstrating that substantial exposure reduction can be achieved without compromising diagnostic utility, including in pediatric and anatomically critical regions [42, 43].

The reduction of parameters such as mA and kVp is the primary strategy for lowering dose in radiographic exams, however, it is known that this reduction can increase image noise and compromise the sharpness of anatomical structures essential for age estimation methods, such as third molars [23]. In this context, optimization should not be interpreted as the pursuit of maximal image quality, but as the identification of exposure conditions that remain diagnostically acceptable for a specific task, in accordance with the ALADAIP principle [22, 23]. In this study, optimization was addressed from a task-based perspective by evaluating whether manufacturer-defined reduced exposure presets remain diagnostically acceptable for forensic age estimation. The reference protocol of 70 kVp and 12.5 mA corresponds to a manufacturer-available preset routinely used for large or adult patients and was employed exclusively as an upper reference condition, following the methodological framework proposed by Martins et al. [25], who demonstrated that substantial dose reductions, including values lower than those recommended for young children, can be achieved without compromising diagnostic performance.

The objective of this study was to evaluate children, adolescents and young adults for the purpose of differentiating individuals younger and older than 18 years, a population that is inherently heterogeneous in terms of craniofacial dimensions. Adolescents close to the legal age threshold frequently present adult-like morphotypes, and the uniform application of the lowest paediatric preset would therefore not be patient-specific. Consequently, the use of a reference benchmark allowed a meaningful assessment of optimization by verifying whether reduced-dose protocols, remain diagnostically reliable for age classification.

To ensure experimental control and reproducibility, acquisitions were performed using manual exposure mode with fixed manufacturer-defined presets, avoiding the uncontrolled variability introduced by automatic exposure control (AEC) systems. This approach, widely adopted in optimization research, allows meaningful assessment of diagnostically acceptable dose reduction by preventing AEC-driven modulation from confounding dose-performance relationships and by enabling exploration of exposure levels below those automatically selected by AEC systems [25, 44–46].

The dichotomous classification of legal majority was assessed only for the I3M method, due to the availability of a validated cutoff value (I3M < 0.08) for distinguishing individuals under and over 18 years of age [36, 47, 48]. High intra-examiner agreement was observed. When interpreted within a clinical context and in light of the dosimetric findings reported by Martins et al. [25], who performed direct dosimetric measurements using the same image acquisition protocols and comparable anthropomorphic phantoms, these results suggest a potential reduction of approximately 77% in the absorbed dose to radiosensitive tissues, such as the salivary glands, with the adjusted acquisition protocol.

This study presents inherent methodological limitations that should be acknowledged. The relatively small sample size, comprising five models and 19 third molars, limits the generalizability of the findings and restricts extrapolation to broader clinical populations. In addition, the analysis focused exclusively on intra-examiner reproducibility, without evaluating the accuracy of age estimation. Future studies should address this question.

## Conclusion

The reduction of exposure parameters in the acquisition of panoramic radiographs does not compromise the reproducibility of the third molar assessment with GHK and I3M techniques. This finding opens new possibilities for safer forensic practices in the field of dental age estimation, including optimal radiation protection for the examination of adolescents and young adults regarding the investigation of legal majority.

## Electronic Supplementary Material

Below is the link to the electronic supplementary material.

Supplementary Material 1 Suppl 1 Methodological flowchart of the study. Schematic representation of the study stages.
