# Supplementary figures and images for: Optimization of panoramic radiographic dose for third molar dental age assessment
Source: Int J Legal Med. 2026 Apr 20;140(4):2313–21. doi: 10.1007/s00414-026-03747-8 (PMC13275526; doi:10.1007/s00414-026-03747-8)

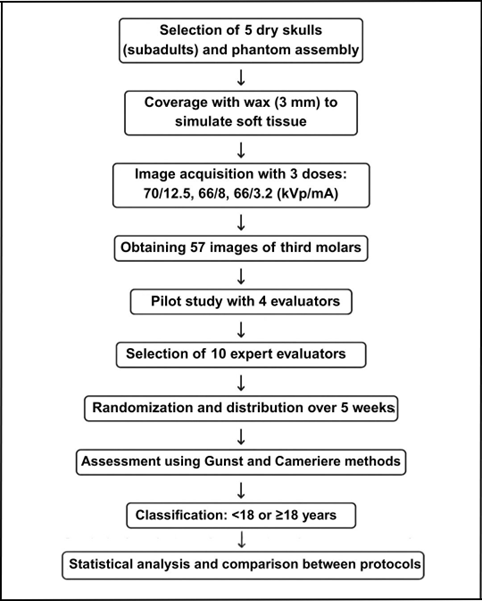


***Suppl 1*** Methodological flowchart of the study. Schematic representation of the study stages.

Supplement: Supplementary file 1 — Supplementary Material 1 Suppl 1 Methodological flowchart of the study. Schematic representation of the study stages. [file 414_2026_3747_MOESM1_ESM.docx]
